# The different damage patterns of short-, middle- and long-range connections between patients with relapse-remitting multiple sclerosis and neuromyelitis optica spectrum disorder

**DOI:** 10.3389/fimmu.2022.1007335

**Published:** 2022-12-02

**Authors:** Xiaoya Chen, Yuling Peng, Qiao Zheng, Dan Luo, Yongliang Han, Qi Luo, Qiyuan Zhu, Tianyou Luo, Yongmei Li

**Affiliations:** Department of Radiology, The First Affiliated Hospital of Chongqing Medical University, Chongqing, China

**Keywords:** multiple sclerosis, neuromyelitis optica spectrum disorder, DTI, short-range connection, middle-range connection, long-range connection

## Abstract

**Objective:**

To investigate the differences in short-, middle- and long-range connections between patients with relapse-remitting multiple sclerosis (RRMS) and neuromyelitis optica spectrum disorder (NMOSD), and their correlation with brain tissue volume, structural and functional network parameters.

**Methods:**

A total of 51 RRMS, 42 NMOSD and 56 health controls (HC) were recruited. Of these 25 RRMS (median: 1.37 years) and 20 NMOSD (median: 1.25 years) patients were also studied at follow-up. The whole-brain fiber connection was divided into three groups according to the trisected lengths of the tract in HC group, including short-, middle- and long-range connections. The brain tissue features (including total brain tissue and deep grey matter volumes) and parameters of DTI and functional networks (including the shortest path, clustering coefficient, local efficiency and global efficiency) were calculated. The differences in fiber number (FN) and average fractional anisotropy (FA) were compared between RRMS and NMOSD by the One-way ANOVA and post hoc tests. The correlation between the FN or FA and the brain tissue volume, DTI and functional network parameters were further analyzed by Pearson analysis.

**Results:**

Compared to HC and NMOSD, the total number of fibers in RRMS was decreased, including the reduced FN of middle- and long-range connections, but increased FN of short-range connections. Compared to HC, the FA of three fibers in RRMS and NMOSD were reduced significantly, and the decrease of FA in RRMS was greater than in NMOSD. There were correlations between the FN of short-, and long-range connections and the atrophy of whole brain tissue in two diseases and structural network topological parameters in RRMS. Additionally, there was no significant difference of FN and FA in short-, middle- and long-range connections between the baseline and follow-up in two diseases.

**Conclusions:**

RRMS and NMOSD patients have different patterns of fiber connection damage. The FN of different lengths in RRMS and NMOSD patients may be associated with brain atrophy. The FN and FA of different lengths may explain the decreased efficiency of the structural network in RRMS patients. In the short-term follow-up, neither has worsened damage of different fibers in two diseases.

## Introduction

Multiple sclerosis (MS) (1) and neuromyelitis optica spectrum disorder (NMOSD) (2) are chronic inflammatory demyelinating diseases of the central nervous system. They differ in pathological and immunological characteristics, but there are similarities and overlaps in the clinical presentation (3). Studies have found that MS and NMOSD patients have focal white matter lesions in conventional MRI and extensive impairment of whole-brain microstructures in diffusion tensor image (DTI) (4–7). Although alterations in white matter fibers can be detected by available MRI techniques, microstructural damage in the white matter fibers remains insufficient to explain the clinical symptoms and neurological impairment in both disorders. At the same time, numerous studies have shown that both MS and NMOSD have large-scale brain network alterations, rather than just local damage of white matter lesions (8, 9).

There are connections of different lengths between different parts of the brain, suggesting that the exchange of information between grey matter and grey matter relies on different lengths of white matter fibers (10, 11). Short-range connecting fibers are more abundant than long-distance connecting fibers, which may connect adjacent anatomical brain gyri and integrate multimodal information (10, 11). Meanwhile, long-range connecting fibers enable effective communication or timely information exchange between different brain regions at a distance (10, 11). However, considering that the morphological features may be the basis of the disease, we wonder whether the fiber damage of different lengths affects the brain morphological changes.

The balance and imbalance between short- and long-range connecting fibers have potential implications for separating and integrating information in human brain networks (11). The different lengths of connections may have other effects on brain networks, implying that damage to either type of connection may have relatively specific effects. Even though there were numerous studies have proved the impairment of brain connection based on graph analysis (12–14), only a few studies have reported that MS patients have varying degrees of impairment in both short- and long-range connections (15, 16). However, it is unclear whether similar or different alteration patterns exist in NMOSD patients as in MS. Additionally, it is unknown whether the damage of different fibers may influence the structural and functional networks in two diseases.

Therefore, in order to further understand the damage patterns of different length fiber and its correlation with the morphology and network efficiency in two different diseases, this study aimed to explore 1) the differences in potential damage to short-, middle- and long-range connecting fibers among relapse-remitting MS (RRMS), NMOSD patients and healthy controls (HC); 2) the correlation between the damage of different fibers and alterations in whole brain volume, structural and functional networks; and 3) the correlation between the damage of different fibers and clinical features.

## Materials and methods

### Participants

In this retrospective study, we recruited 51 RRMS patients, 42 NMOSD patients and 56 HC between October 2014 and September 2019. The inclusion criteria were as follows: (1) Patients with RRMS diagnosed according to the 2017 revised McDonald criteria (17), and NMOSD patients according to the revised NMOSD diagnostic criteria (18); (2)18-60years; (3) All patients in relapse-free state and not taking drugs such as high-dose steroids for at least 2 weeks before MRI scanning. The exclusion criteria were as follows: (1) patients with contraindications for MRI scans and other neurological or psychiatric diseases, (2) image artifacts or incomplete clinical information. Among 42 NMOSD patients, 69% (29/42) were AQP4 antibody-positive for serology and cerebrospinal fluid status and 31% (13/42) were negative measured by an indirect immunofluorescence method. The Institutional Review Board approved the study protocol of the First Affiliated Hospital of Chongqing Medical University, and the subjects have provided written informed consent. The cohort has been described in previous publications (19), but fiber length characteristics were not investigated. All participants recorded demographic and clinical information. Additionally, the extended disability status score (EDSS) was used to assess overall disability.

Twenty-five RRMS patients and 20 NMOSD patients in a cross-sectional cohort were followed. The MRI scan interval between two scans in each patient ranged from 0.58 to 5.67 years (median, 1.67 years). Among 20 NMOSD patients, 70% (14/20) were AQP4 positive and 30% (6/20) were negative. All RRMS and NMOSD patients at follow-up had received only steroid therapy, and none had received any disease-modifying treatment.

### MRI image acquisition

MRI was performed on a 3T system (GE Healthcare, Milwaukee, USA). All the participants underwent conventional brain MRI acquisition for lesion detection, including axial 2D T1-weighted imaging (repetition time (TR) 200ms, echo time (TE) 2.86ms and 25 slices), 2D T2-weighted imaging (TR 3600 ms, TE 120 ms and 25 slices) and 2D fluid-attenuated inversion recovery (TR 8000 ms, TE 120 ms and 25 slices). In addition, for the quantitative analysis, the MRI protocol consisted of a high-resolution 3D T1 sequence (TR 8300 ms, TE 3.3 ms, isotropic voxels of dimension 1 mm^3^, 156 slices), a diffusion tensor imaging (DTI) (TR 5700 ms, TE 86 ms, voxel size 2.5×2.5×2.3 mm^3^, 53 slices, 4 b-values of 0 and 34 diffusion directions with b-values of 1000s/mm^2^), and a resting-state blood oxygen level-dependent (BOLD) sequence was acquired (TR 2000 ms, TE 30 ms, contiguous axial slices 3.0 mm, 240 volumes and 33 slices).

### Lesion volume

For RRMS and NMOSD patients, T2WI lesion volumes were obtained automatically using the lesion segmentation tool (LST) (20) and SPM12 software. Then the lesions mask in patients were used to fill 3D T1-weighted MR images for next process by lesion fill tool (LFT) (21).

### T1 imaging preprocesses

The automated processing of whole brain tissue segmentation was calculated from the 3D T1-weighted MR images using FreeSurfer software version 6.0 (https://surfer.nmr.mgh.harvard.edu/fswiki/). The morphology of the whole brain was calculated by the volume of 14 brain features (bilateral cerebellar cortex, bilateral cerebellar white matter, bilateral cerebral cortex, bilateral cerebral white matter, brain stem, total cortex, total cerebral white matter, total deep grey matter, total grey matter and total intracranial volume). The deep grey matter was divided into 16 deep grey matter brain regions (bilateral thalamus, bilateral caudate nucleus, bilateral putamen, bilateral globus pallidus, bilateral amygdala, bilateral nucleus accumbens, bilateral ventral diencephalon and bilateral hippocampal)(Supplementary Material).

### Fiber analysis

**Figure 1:**
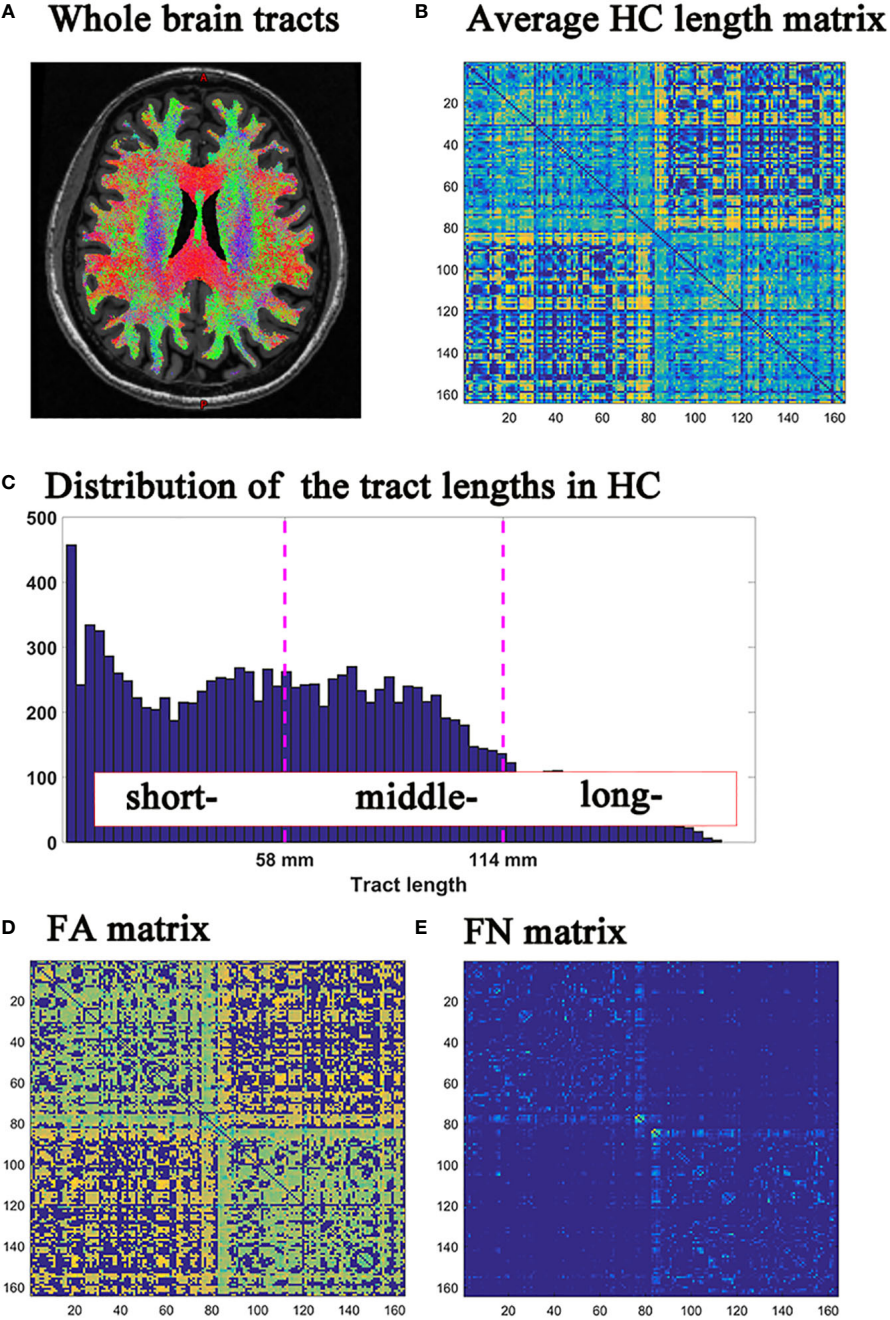
**(A)** Whole brain tracts; **(B)** HC group average fiber length matrix; **(C)** Histograms of fiber length distribution in HC group; **(D)** FN matrix; **(E)** FA matrix. HC, healthy controls; FN, fiber number; FA, fractional anisotropy.

Analysis of DTI data was performed using FSL software and MRtrix software (22) (https://www.mrtrix.org). The DTI preprocesses included dwidenoise, mrdegibbs, eddy_correct, dwibiascorrect, 5ttgen, dwitensor and tensor2metric. Next, whole brain fiber tracking was performed: dwi2response was used to calculate the response function; dwi2fod was used to evaluate the direction of the fiber bundle; tckgen was used to track the probabilistic fiber in the whole brain. (**Figure 1A**) Then, the whole brain connections (including fiber length matrix, fiber number (FN) matrix, and fractional anisotropy (FA) matrix) were calculated by tck2connectome, with the nodal definition based on the 148 grey matter from Destrieux-atlas in Freesurfer software (23).

The histogram of tract lengths in the HC group was based on the average fiber lengths between each brain region area in HC. (**Figure 1B**) The fiber lengths of the HC group were trisected according to the minimum and maximum values of the tract lengths histogram (**Figure 1C**). The fiber lengths of the three groups were shown in Supplementary Material (**eFigure 1**).

Further, the fibers of all subjects in HC, MS and NMOSD groups were classified into short-, middle- and long-range connections, based on the fiber lengths obtained by trisection in HC group as previously describe (15). In order to reflect the degree of brain damage, the fiber number (FN) was used to quantify macrostructural properties, and the average fractional anisotropy (FA) was used to assess microstructural integrity (**Figures 1D, E**).

### Structural and functional network

Structural networks were constructed by MRtrix3 software and functional networks were constructed by GRETNA software in all participants. The incorporated topological property metrics included: the shortest path, clustering coefficient, local efficiency and global efficiency, (Supplementary Material).

### Statistical analyses

The age, duration, EDSS and lesion volumes were analyzed using the Kolmogorov-Smirnov test for normality in Prism software (version 8.0.1). The One-way ANOVA and *post hoc* tests or independent-sample t-test was used for data conforming to the normal distribution. The Wilcoxon or non-parametric Kruskal-Wallis test was used for data with non-normal distribution. The sex data were analyzed with the chi-square test.

The One-way ANOVA and post hoc tests were used to compare the differences in FN and FA for short-, middle- and long-range connections in HC, RRMS and NMOSD groups, with age and sex as covariates for the three groups. The correlation analysis between the damage of short-, middle- and long-range connections and total brain tissue volume, structural and functional network topological parameters, and clinical parameters were analyzed using Pearson or Spearman. The *p*<0.05 was considered to be a statistically significant difference after false discovery rate (FDR) correction for multiple comparisons. The effect size was then calculated using Cohen’s d. To examine whether the FN and FA for short-, middle- and long-range connections might serve as potential biomarkers for differentiating the patients with MS and NMOSD, the receiver operating characteristic (ROC) curve was plotted by 5-fold cross-validations. This procedure was performed using the scikit-learn codes (https://scikit-learn.org/0.22/modules/generated/sklearn.metrics.roc_auc_score.html).

Paired t-tests were performed on the baseline and follow-up of the RRMS group and the NMOSD group, respectively.

## Results

### Demographic characteristics, neuropsychological and conventional MRI data

In the cross-sectional cohort, the demographic, clinical and neuropsychological characteristics of RRMS, NMOSD and HC are summarized in **Table 1**. However, the number of female patients was higher in NMOSD group (39/3) than in HC (31/25) and RRMS (32/19) groups. There were no significant differences in age between the three groups and in disease duration and EDSS between the two diseases. The T2-lesion volume in RRMS was higher than in NMOSD (**Table 1**)

**Table 1:** Demographic, clinical and neuropsychological variables at cross-sectionally study.

	HC	RRMS	NMOSD	*p* value	*p* value	*p* value
				HC vs. RRMS	HC vs. NMOSD	RRMS vs. NMOSD
age (Y)	37.38 ± 14.29	40.67 ± 14.08	40.48 ± 13.70	0.444^a^	0.355^a^	0.972^a^
sex (F/M)	31/25	32/19	39/3	0.561^c^	**<0.001^c^**	**0.001^c^**
duration (Y)	–	5.50 (7.75)	3.00 (6.50)	–	–	0.130^b^
EDSS	–	2.50 (2.00)	3.00 (1.50)	–	–	0.120^b^
T_2_ lesion volume (cm^3^)	–	17.98 ± 13.45	3.69 ± 8.57	–	–	**<0.001^b^**

Data are represented as mean ± standard deviation or median (interquartile range). HC, healthy controls; RRMS, relapse-remitting multiple sclerosis; NMOSD, neuromyelitis optica spectrum disorder; F: female; M: male; Y: years; EDSS, expanded disability status scale; a: one-way ANOVA and post-hoc analysis; b: Wilcoxon test; c: chi-square test; bold text: significant difference.

In the longitudinal cohort, there were no significant differences in age and the time interval between baseline and follow-up in patients with NMOSD and RRMS, while the ratio of females to males was higher in NMOSD than in RRMS. Both RRMS and NMOSD had higher EDSS and T_2_WI lesion volume in follow-up than baseline (**Table 2**)

**Table 2:** Demographic and clinical variables at longitudinal study.

	RRMS		*p value*	NMOSD		*p value*	*p value*
	baseline	follow-up	RRMS baseline vs. follow-up	baseline	follow-up	RRMS baseline vs. follow-up	RRMS baseline vs. NMOSD baseline
age (y)	38.8 ± 11.11	–	–	41 ± 13.78	–	–	**0.556^a^**
sex (f/m)	14/11	–	–	19/1	–	–	**0.005^d^**
interval (y)	–	1.37(1)	–	–	1.25(1.8)	–	0.826^c^
EDSS	1.86 ± 1	2.25 ± 1.22	**0.001^b^**	2.25 ± 1.22	3.4 ± 1.71	**0.017^b^**	–
T_2_WI lesion volume (cm^3^)	15.63(34.22)	17.12(28.67)	**0.026^c^**	1.21(4.32)	2.22(4.60)	**0.013^c^**	–

Data are represented as mean ± standard deviation or median (interquartile range). RRMS, relapse-remitting multiple sclerosis; NMOSD, neuromyelitis optica spectrum disorder; EDSS, Expanded Disability Status Scale; a: independent-sample t-test; b: paired-test, c: Wilcoxon test; d: chi-square test; bold text: significant difference.

### Damage to short-, middle- and long-range connections

In general, all three groups showed that long-range connections have the lowest FN, followed in order by short-, and middle-range connections; and in terms of FA, long-range connections have the highest FA, followed by middle-, and short-range connections.

**Figure 2:**
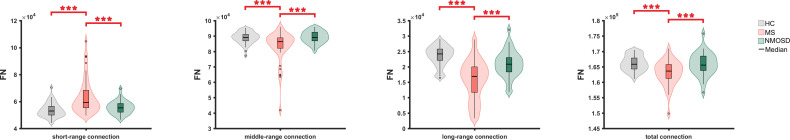
The FN comparison of short-, middle-, long- and total fiber connections between three groups. FN, fiber number ; *: <0.05, **<0.01, ***<0.001.

Compared to NMOSD and HC, the total number of fibers in RRMS was reduced, with a reduction in the FN of middle- and long-range connections (*p*<0.05, FDR corrected) and an increase in the FN of short-range connections (*p*<0.05, FDR corrected). And the FN was more severely reduced in long-range connections than in middle-range connections (**Figure 2**)

**Figure 3:**
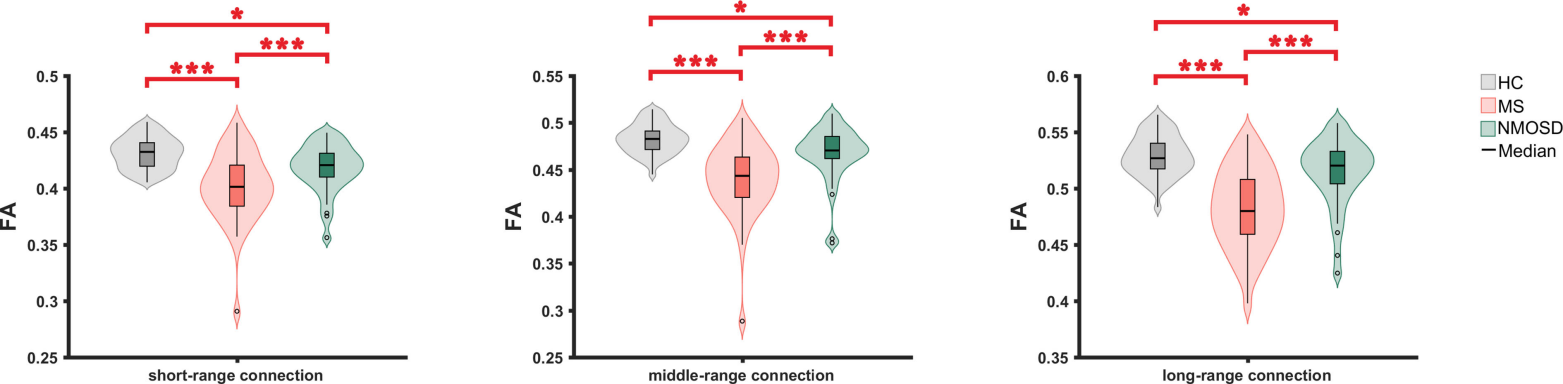
The FA comparison of short-, middle- and long connections between three groups. FN, fractional anisotropy ; *: <0.05, **<0.01, ***<0.001.

The microstructural metrics of short-, middle- and long-range connections were further analyzed in three groups. Compared to HC, the FA of all three connections was significantly reduced in RRMS and NMOSD (*p*<0.05, FDR corrected). Moreover, the difference in the decreased FA between RRMS and NMOSD was significant (*p*<0.05, FDR corrected). The degree of the reduction in FA for all three different lengths of connections was greater in RRMS than in NMOSD, suggesting a more severe impairment of white matter integrity in RRMS (**Figure 3**)

**Figure 4:**
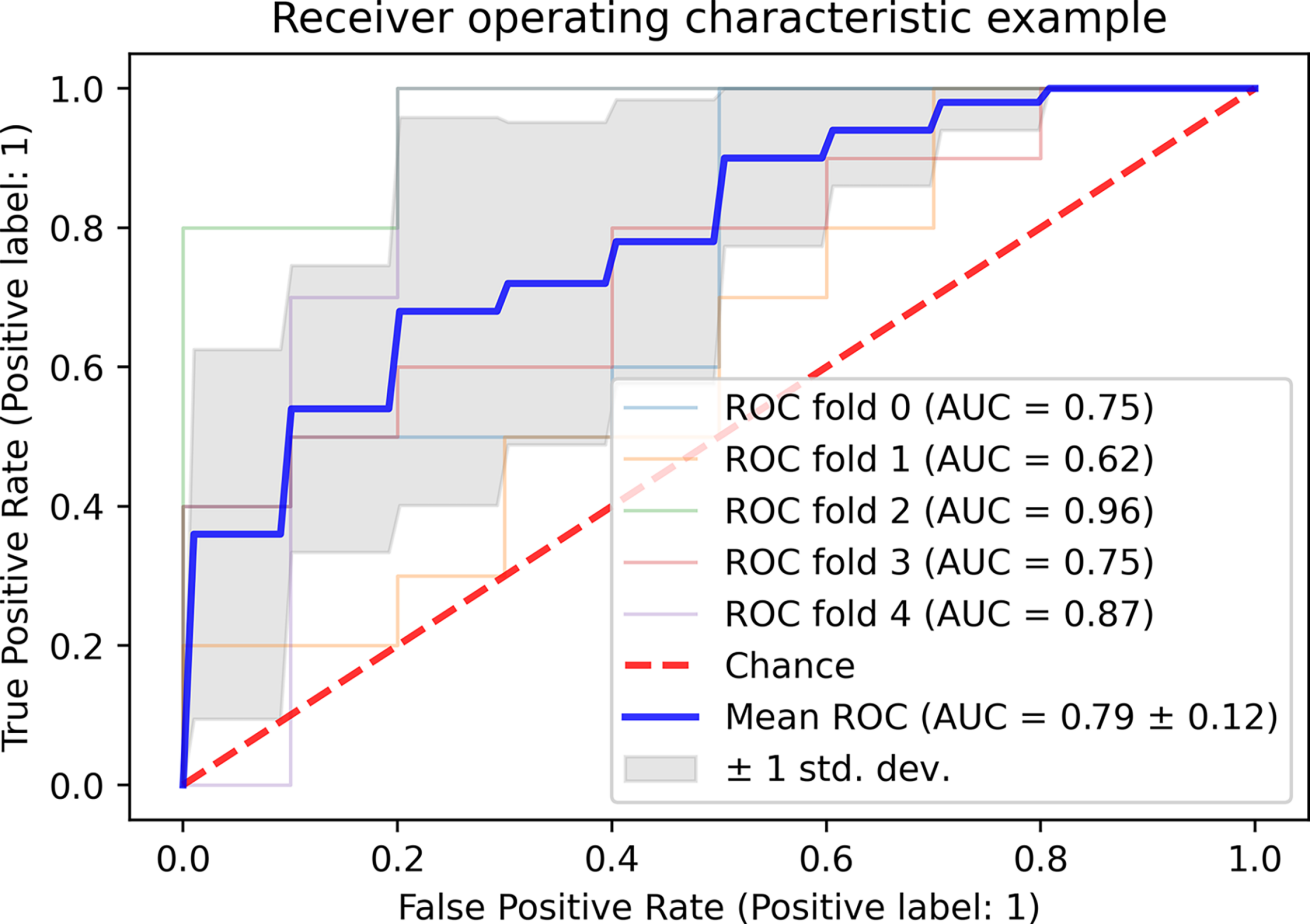
The classification of the patients with MS and NMOSD based on FN and FA of short-, middle- and long fiber. MS, multiple sclerosis; NMOSD, neuromyelitis optica spectrum disorder; FN: fiber number; FA, fractional anisotropy; ROC, receiver operator characteristic curve; AUC, area under curve.

The FN and FA for short-, middle- and long-range connections exhibited the highest power for distinguishing the patients with MS from the patients with NMOSD (mean area under curve = 0.79 ± 0.12) (**Figure 4**). The corresponding relationships between the two features were shown in Supplementary Material (**eFigure 2**).

The result of damage to short-, middle-, long-range connections, global brain volume, deep grey matter were summarized in **eTable 1** and **eTable 2**.

### Correlations with global brain volume

In patients with RRMS, the volumes of 14 brain tissue features were negatively correlated with the FN of short-range connections, the volumes of 13 brain tissue features were positively correlated with the FN of long-range connections and the volumes of 12 brain tissue features were positively correlated with the FN of middle-range connections, including bilateral cerebellar cortex, cerebellar white matter, cerebral cortex, cerebral white matter, brainstem, total cortex, total white matter, deep grey matter, total grey matter and total intracranial volume. In patients with NMOSD, the volumes of 9 brain tissue features were negatively correlated with the FN of short-range connections, and the volumes of 7 brain tissue features were positively correlated with the FN of long-range connections, including bilateral cerebellar cortex, cerebellar white matter, cerebral cortex, brainstem, total white matter and total grey matter (**Table 3**)

**Table 3:** The correlation between FN of different fibers connections lengths and the total brain tissue volume in RRMS and NMOSD.

FN	RRMS						NMOSD					
	short-range		middle-range		long-range		short-range		middle-range		long-range	
	*p* value	r value	*p* value	r value	*p* value	r value	*p* value	r value	*p* value	r value	*p* value	r value
L cerebellar cortex	**0.01**	-0.363	**-**	–	**-**	–	**0.003**	-0.459	–	–	**-**	–
R cerebellar cortex	**0.013**	-0.352	**-**	–	**<0.001**	0.509	**0.007**	-0.426	–	–	**-**	–
L cerebellar white matter	**<0.001**	-0.486	**0.011**	0.361	**0.001**	0.45	**<0.001**	-0.594	–	–	**0.001**	0.533
R cerebellar white matter	**0.001**	-0.476	**0.015**	0.347	**0.021**	-0.329	**0.001**	-0.524	–	–	**0.002**	0.487
L cerebral cortex	**<0.001**	-0.652	**0.001**	0.495	**<0.001**	0.538	**<0.001**	-0.631	–	–	**<0.001**	0.668
R cerebral cortex	**<0.001**	-0.602	**0.001**	0.445	**<0.001**	0.494	**<0.001**	-0.63	–	–	**<0.001**	0.682
L cerebral white matter	**<0.001**	-0.731	**0.001**	0.511	**<0.001**	0.701	**-**	–	–	–	**-**	–
R cerebral white matter	**<0.001**	-0.703	**0.001**	0.46	**<0.001**	0.666	**-**	–	–	–	**-**	–
brainstem	**<0.001**	-0.625	**0.004**	0.4	**<0.001**	0.563	**0.001**	-0.55	–	–	**0.006**	0.432
total cortex	**<0.001**	-0.633	**0.001**	0.483	**<0.001**	0.517	**-**	–	–	–	**-**	–
total white matter	**<0.001**	-0.719	**0.001**	0.484	**<0.001**	0.68	**<0.001**	-0.628	–	–	**<0.001**	0.68
total deep grey matter	**<0.001**	-0.682	**0.001**	0.462	**<0.001**	0.663	**-**	–	–	–	**-**	–
total grey matter	**<0.001**	-0.621	**0.001**	0.468	**<0.001**	0.518	**0.004**	-0.447	–	–	**<0.001**	0.69
total intracranial volume	**0.001**	-0.482	**-**	–	**0.015**	0.346	**-**	–	–	–	**-**	–

FN, fiber number; L, left; R, right.; bold text: significant difference.

There was no statistically significant correlation between brain tissue volume and mean FA of three different lengths of connections in both RRMS and NMOSD.

In RRMS, the volumes of 16 deep grey matter were negatively correlated with the FN of short-range connections and positively correlated with the FN of long-range connections, and the volumes of 14 deep grey matter were positively correlated with the FN of middle-range connections, including the bilateral thalamus, caudate nucleus, nucleus accumbens, pallidum, hippocampus, amygdala, nucleus ambiguous and ventral mesencephalon. In NMOSD, left hippocampal volume was negatively correlated with the FN of short-range connections (r=-0.43, *p*=0.006), and three deep grey matter volumes were positively correlated with the FN of long-range fibers, including the right thalamus (r=0.44, *p*=0.005), left hippocampus (r=0.416, *p*=0.008) and right ventral septal nucleus (r=0.404, *p*=0.011) (**Table 4**)

**Table 4:** The correlation between FN of different fibers connections lengths and the deep grey matter volume in RRMS and NMOSD.

FN	RRMS						NMOSD					
	short-range		middle-range		long-range		short-range		middle-range		long-range	
	*p* value	r value	*p* value	r value	*p* value	r value	*p* value	r value	*p* value	r value	*p* value	r value
L thalamus	**<0.001**	-0.624	**0.001**	0.453	**<0.001**	0.608	–	–	–	–	**-**	–
R thalamus	**<0.001**	-0.72	**<0.001**	0.526	**<0.001**	0.699	–	–	–	–	**0.005**	0.44
L caudate nucleus	**<0.001**	-0.549	**0.022**	0.328	**<0.001**	0.503	–	–	–	–	**-**	–
R caudate nucleus	**<0.001**	-0.549	**0.012**	0.356	**<0.001**	0.541	–	–	–	–	**-**	–
L putamen	**<0.001**	-0.656	**<0.001**	0.5	**<0.001**	0.64	–	–	–	–	**-**	–
R putamen	**<0.001**	-0.682	**<0.001**	0.488	**<0.001**	0.644	**-**	–	–	–	**-**	–
L pallidum	**<0.001**	-0.5	**0.011**	0.362	**0.005**	0.399	**-**	–	–	–	**-**	–
R pallidum	**<0.001**	-0.57	**0.015**	0.344	**<0.001**	0.555	**-**	–	–	–	**-**	–
L hippocampus	**<0.001**	-0.517	**0.01**	0.364	**<0.001**	0.511	**0.006**	-0.43	–	–	**0.008**	0.416
R hippocampus	**<0.001**	-0.505	**0.013**	0.354	**<0.001**	0.513	**-**	–	–	–	**-**	–
L amygdala	**0.009**	-0.372	**-**	–	**0.001**	0.445	**-**	–	–	–	**-**	–
R amygdala	**0.011**	-0.362	**-**	–	**0.007**	0.381	**-**	–	–	–	**-**	–
L nucleus accumbens	**<0.001**	-0.646	**0.001**	0.456	**<0.001**	0.644	**-**	–	–	–	**-**	–
R nucleus accumbens	**<0.001**	-0.551	**0.001**	0.482	**<0.001**	0.524	**-**	–	–	–	**0.011**	0.404
L ventral diencephalon	**<0.001**	-0.586	**0.002**	0.441	**<0.001**	0.555	**-**	–	–	–	**-**	–
R ventral diencephalon	**<0.001**	-0.603	**0.001**	0.461	**<0.001**	0.55	**-**	–	–	–	**-**	–

FN, fiber number; L, left; R, right; bold text: significant difference.

Bilateral caudate nucleus volumes in RRMS were positively correlated with mean FA of long-range connections (L: r=0.382, *p*=0.007; R: r=0.379, *p*=0.007). There was no statistically significant correlation between brain tissue volume and mean FA of three different lengths of connections in NMOSD.

### Correlations with structural network efficiency

For RRMS, the shortest path length was positively correlated with the FN of short-range connections (r=0.493, *p*=0.001) and negatively correlated with the FN of middle- and long-range connections (r=-0.568, *p*=0.001; r=-0.646, *p*<0.001, respectively), the global efficiency was negatively correlated with the FN of short-range connections (r=-0.493, *p*=0.001) and positively correlated with the FN of middle- and long-range connections (r=0.568, *p*=0.001; r=0.646, *p*<0.001, respectively), and the clustering coefficient and local efficiency were positively correlated with the FN of long-range connections (r=0.359, *p*=0.011; r=0.362, *p*=0.011, respectively). No statistically significant correlation was observed between the DTI structural network efficiency and the FN of short-, middle- and long-range connections in NMOSD.

In RRMS, the shortest path length was negatively correlated with the mean FA of short-, middle- and long-range connections (r=-0.49, *p*<0.001; r=-0.502, *p*<0.001; r=-0.572, *p*<0.001, respectively), the global efficiency was positively correlated with the mean FA of short-, middle- and long-range connections (r=0.49, *p*<0.001; r=0.502, *p*<0.001; r=0.572, *p*<0.001, respectively), and the clustering coefficient and local efficiency were positively correlated with the mean FA of long-range connections (r=0.408, *p*=0.004; r=0.413, *p*=0.003, respectively). In NMOSD, there was no statistically significant correlation between the DTI structural network connectivity and the mean FA of connections.

### Correlations with the functional brain network

There was no statistically significant correlation between the FN as well as the mean FA of short-, middle- and long-range connections and functional network topological metrics for both RRMS and NMOSD.

### Correlations with lesion volume and clinical parameters

In patients with RRMS, negative correlations were observed between T2WI lesion volume and FN (r=-0.32, *p*=0.028) and FA (r=-0.513, *p*<0.001) of long-range connections. The sleep scale was negatively correlated with the FN of short-range connections (r=-0.48, *p*=0.011) and positively correlated with the FN of long-range connections (r=0.455, *p*=0.023).

No correlation was observed between the clinical parameters and FN and FA of connections in NMOSD.

### Comparison results of longitudinal cohort groups

In RRMS and NMOSD groups, there was no significant difference of FN and FA in short-, middle- and long-range connections between the baseline and follow-up.

## Discussion

In this study, probabilistic tractography was performed on whole-brain fiber tracts. The whole-brain fibers were classified into short-, middle- and long-range connections according to the length of the fibers in HC group. The differences in connections damage of different lengths between RRMS and NMOSD, and their correlation with whole-brain tissue volume, structural network and functional network efficiency were investigated. RRMS patients had a significant reduction in the number of total fibers, including a significant reduction in FN for middle- and long-range connections and an increase in FN for short-range connections, as well as a severe loss of tract integrity (i.e. significant reduction in FA) for all three types of connections. In contrast, NMOSD patients were found to have only moderate decreases in fractional anisotropy of short-, middle- and long-range connections, with a lesser loss of tract integrity than RRMS patients. All of these features showed good performance for differentiating RRMS patients from NMOSD patients. Further correlation analyses revealed a broad correlation between atrophy of whole-brain structures, the efficiency of structural networks and short-, and long-range connections in RRMS and NMOSD.

The reduced FN in middle- and long-range connections and increased FN in short-range connections were shown in RRMS patients, as well as reduced FA in short-, middle- and long-range connections. The multiple demyelinating lesions around the ventricles in MS patients are prone to damage to long-range connections. It may be helpful to explain that long-range connections are more likely to undergo damage in MS (24, 25). At the same time, a previous study also reported that early MS is more likely to have short-range connection damage (16). Thus, pathological changes, such as specific white matter demyelination, axonal damage and degenerative degeneration, may cause the presence of extensive white matter damage and microstructural damage of different lengths of connections in MS patients.

Additionally, we found no significant changes in the FN of the three connections in NMOSD patients, and only a mild reduction in FA, suggesting that there is substantial microstructural damage to all three connections, but to a lesser extent. It is consistent with the results of previous studies that found mild microstructural damage in NMOSD (5, 19). The results of this study suggest that the damage pattern of the three different length connections in NMOSD patients differs from that of MS patients, possibly because of the different pathological processes that exist in NMOSD patients compared to MS patients, and also laterally reflect the lesser degree of underlying pathological changes in NMOSD (26).

Moreover, we showed that the FN and FA in different lengths of fiber exhibited good performance for differentiating the patients with MS from NMOSD. Given that MS patients showed more white matter damage compared with NMOSD, these findings are particularly encouraging because different damage patter of different fibers may provide promising ways for objective diagnosis between MS and NMOSD. Notably, the preliminary classification findings were derived from the linear discriminant analysis given the relatively small sample size. Future studies should cross-validate the generalization of our findings by using a large sample size.

We found that the volumes of whole-brain and deep grey matter were positively correlated with FN of middle- and long-range connections in RRMS, but negatively correlated with FN of short-range connections. It may indicate that underlying white matter pathological alterations contribute to the atrophy of brain tissue in MS (e.g., direct connections of white matter fibers to grey matter may lead to potential retrograde damage) (24). In NMOSD patients, we found that brain tissue volumes and deep grey matter volumes were positively correlated with the FN of long-range connections and negatively correlated with the FN of short-range connections. It may suggest that NMOSD has a partially similar damage pattern to RRMS, but fewer correlations exist than RRMS. Therefore, we consider that damage to long- and short-distance connections may be generalizable to brain atrophy.

The integrity of structural network connections is reduced in people with RRMS, and the reduced brain network efficiency is positively correlated with FN for middle- and long-range connections, but negatively correlated with FN for short-range connections. The different regions of the brain communicate with each other *via* long-distance connecting fibers. Therefore, the long-distance connecting fibers are considered fast pathways for network topology, facilitating information exchange between distant brain regions and maintaining the efficiency of brain networks (15). In MS, the more severe the disruption of middle- and long-distance connections, the greater the loss of efficiency of the structural brain network is suggested (27). In contrast, an increase in FN of short-range connections indicates a shift in the balance of brain networks from the dominance of long-range connections to control of short-range connections. Due to severe damage to middle- and long-distance connections, the networks under their control cannot maintain the efficient functioning of the brain. Therefore, in order to maintain the balance of brain networks, an increase in short-range connections may enhance the transmission of information. On the other hand, there was no correlation observed between reduced brain network efficiency and alterations in fibers of different lengths in people with NMOSD.

In general, consistent with the previous studies, the damage to long-range connections was predominantly related to a less efficient network organization in multiple sclerosis patients (15, 28). The connection of long-distance connecting fibers is treated as a relatively expensive long connection, which is very important for integrating the information of different parts of the brain. When long-distance fibers are damaged in the brain, it is easier to reduce brain efficiency, resulting in damage to information communication in the brain. As for the middle connection, we believe that it may play a similar role to long-distance connection, because it also showed positive correlations with reduced network efficiency. On the contrary, the previous study found that there is an imbalance between short- and long-range connectivity in the efficiency of brain networks, which may further change the efficiency of structural networks (15). Our study further confirms that there were negative correlations between short-distance connections and network efficiency. Different from the structural network, the functional network failed to find any correlation between the fibers with different lengths. This may be related to the more complex mechanism behind the functional network, which needs to be further studied in future research.

In addition, the longitudinal differences in short-, middle- and long-range connections of small samples were also discussed in this study. The results showed that neither RRMS nor NMOSD patients showed significant changes in the short-term follow-up, which may be related to the short follow-up time or the slight aggravation of the disease status of patients during the follow-up period.

There are several limitations of the present study. Firstly, the number of patients in this study was relatively small. Secondly, there is the negative result of our longitudinal part, which is needed to further verify by an enlarged sample size and longer follow-up. Thirdly, future studies could further explore the differences in damage to connections of different lengths in white matter lesions and normal-appear whiter matter, and their relationship to pathology. Fourthly, this study did not further investigate the corresponding brain area of different length fibers, and it is meaningful to find the underlying pathological foundation.

## Conclusion

RRMS and NMOSD have different patterns of fiber connection damage. Both RRMS and NMOSD patients had microstructural damage in short-, middle- and long-range connections. However, RRMS patients showed specifically reduced FN of middle- and long-distance connections, whereas NMOSD patients had no alterations in FN of the three different connections. Alterations in all three different lengths of fibers in RRMS and NMOSD patients may be associated with brain atrophy. At the same time, the pattern of damage to fiber connections in RRMS also leads to a reduced ability for information integration in structural networks. Based on the different lengths of fibrous connections, combined with morphological and topological network structural analysis, this study provides a potentially comprehensive explanation for the pathological changes in RRMS and NMOSD patients.

## Data availability statement

The original contributions presented in the study are included in the article/**Supplementary Material**. Further inquiries can be directed to the corresponding authors.

## Ethics statement

The studies involving human participants were reviewed and approved by The Institutional Review Board of the First Affiliated Hospital of Chongqing Medical University. The patients/participants provided their written informed consent to participate in this study. Written informed consent was obtained from the individual(s) for the publication of any potentially identifiable images or data included in this article.

## Author contributions

XC and YP collected and analyzed the data. XC, TL and YL designed the study, reviewed and edited the manuscript. All authors contributed to the article and approved the submitted version.

## Funding

This study has received funding by the Key Project of Technological Innovation and Application Development of Chongqing Science and Technology Bureau (CSTC2021 jscx-gksb-N0008).

## Acknowledgments

All authors would like to thank all the subjects who participated in this study.

## Conflict of interest

The authors declare that the research was conducted in the absence of any commercial or financial relationships that could be construed as a potential conflict of interest.

## Publisher’s note

All claims expressed in this article are solely those of the authors and do not necessarily represent those of their affiliated organizations, or those of the publisher, the editors and the reviewers. Any product that may be evaluated in this article, or claim that may be made by its manufacturer, is not guaranteed or endorsed by the publisher.

## Supplementary material

Supplementary Figure 1 The distribution of short-, middle-, and long-range connection in HC, RRMS and NMOSD patients.

Supplementary Figure 2 The relationships about FN and FA of short-, middle- and long-range connection in patients with RRMS and NMOSD. FN, fiber number; FA, fractional anisotropy.
