# Supplementary material for: The different damage patterns of short-, middle- and long-range connections between patients with relapse-remitting multiple sclerosis and neuromyelitis optica spectrum disorder
Source: Front Immunol. 2022 Dec 2;13:1007335. doi: 10.3389/fimmu.2022.1007335 (PMC9755727; doi:10.3389/fimmu.2022.1007335)
Supplement: Supplementary file 1 [file DataSheet_1.docx]

Supplementary Material

# 3DT1 data processing

The recon-all code in FreeSurfer 6.0 software was used to segment all brain tissue (https://surfer.nmr.mgh.harvard.edu/fswiki/). The volume of the lateral choroid plexus was calculated. According to the Destrieux-atlas in FreeSurfer software. The whole brain was calculated by the volume of 14 total brain tissue (bilateral cerebellar cortex, bilateral cerebellar white matter, bilateral cerebral cortex, bilateral cerebral white matter, brain stem, total cortex, total cerebral white matter, total deep grey matter, total grey matter and total intracranial volume). The deep grey matter was divided into 16 deep grey matter brain regions (bilateral thalamus, bilateral caudate nucleus, bilateral putamen, bilateral globus pallidus, bilateral amygdala, bilateral nucleus accumbens, bilateral ventral diencephalon and bilateral hippocampal) were included.

**Structure network**

Structural networks were constructed by MRtrix3 software based on the probabilistic tractography. Based on aparc-a2009s template in Freesurfer, the nodes were defined as 164 brain regions, including 74 cortical brain regions bilaterally and 8 deep grey matter. Each edge was defined as the value of the number of fiber tracts between each two brain regions divided by the volume of the two brain regions. A final DTI network matrix of 164×164 was generated. The generated DTI networks were analyzed for topological properties based on the GRETNA toolkit. The incorporated topological property metrics included: the shortest path, clustering coefficient, local efficiency, global efficiency, rich club connectivity, feeder connectivity, and peripheral connectivity.

**Functional network**

The resting-state functional MRI data (BOLD) were preprocessed based on SPM12 (http://www.fil.ion.ucl.ac.uk/spm/software/spm12/) and the GRETNA Statistical analyses. The main components include: (i) removal of the first 10 time points; (ii) slice timing correction; (iii) motion correction; (iv) spatial normalization to the Montreal Neurological data, with the help of Freesurfer's algorithm for inter-modal alignment (https://surfer.nmr.mgh.harvard.edu/fswiki/bbregister). (5) Smoothing: the normalized functional data images were smoothed with a Gaussian kernel (half height, full width = 4 mm); (6) Filtering; (7) Regression noise; (8) Scrubbing. subjects with head movements larger than 3 mm or 3° were excluded.

The binarized functional network matrix is constructed based on GRETNA extracting the time series from the pre-processed functional data. The nodes were set to 90 brain regions according to the AAL template. Each edge was defined as the value of the time series correlation coefficient between every two brain regions. And Z-transformation is performed on the generated network matrix values to finally generate a 90 × 90 functional connectivity matrix. The topological properties of the generated binarized network were analyzed based on the GRETNA, where the values of sparsity were categorized as 0.01-0.5 with a step size of 0.01. The functional network incorporated topological property metrics including: the shortest path, clustering coefficient, local efficiency, global efficiency, rich club connectivity, feeder connectivity, and peripheral connectivity.

eFig 1. The distribution of short-, middle-, and long-range connection in HC, RRMS and NMOSD patients.

eFig 2. The relationships about FN and FA of short-, middle- and long-range connection in patients with RRMS and NMOSD. FN, fiber number; FA, fractional anisotropy.

eTable 1. The difference of FN and FA between HC, RRMS and NMOSD patients.

|  | HC-RRMS | | | HC-NMOSD | | | RRMS-NMOSD | | |
| --- | --- | --- | --- | --- | --- | --- | --- | --- | --- |
|  | *p* value | t value | cohen's d | *p* value | t value | cohen's d | *p* value | t value | cohen's d |
| FN |  |  |  |  |  |  |  |  |  |
| short-range | <0.001 | -5.834 | -1.03 | 0.855 | -0.183 | -0.402 | <0.001 | 5.091 | 0.812 |
| middle-range | <0.001 | 3.864 | 0.667 | 0.569 | -0.571 | -0.103 | <0.001 | -4.068 | -0.648 |
| long-range | <0.001 | 8.529 | 1.633 | 0.067 | 1.843 | 0.736 | <0.001 | -5.862 | -1.006 |
| total-connection | <0.001 | 3.371 | 0.579 | 0.952 | 0.06 | 0.099 | 0.003 | -2.987 | -0.5 |
| FA |  |  |  |  |  |  |  |  |  |
| short-range | <0.001 | 6.79 | 1.311 | 0.012 | 2.542 | 0.8 | <0.001 | -3.579 | -0.641 |
| middle-range | <0.001 | 7.589 | 1.539 | 0.016 | 2.427 | 0.711 | <0.001 | -4.417 | -0.803 |
| long-range | <0.001 | 8.636 | 1.806 | 0.021 | 2.328 | 0.653 | <0.001 | -5.474 | -1.017 |

Note: HC, healthy controls; RRMS, relapse-remitting multiple sclerosis; NMOSD, neuromyelitis optica spectrum disorder; FN, fiber number; FA, fractional anisotropy

eTable 2. The difference of brain features and deep grey matter between HC, RRMS and NMOSD patients.

|  | HC-RRMS | | | HC-NMOSD | | | RRMS-NMOSD | | |
| --- | --- | --- | --- | --- | --- | --- | --- | --- | --- |
|  | *p* value | t value | cohen's d | *p* value | t value | cohen's d | *p* value | t value | cohen's d |
| **Brain features** |  |  |  |  |  |  |  |  |  |
| Left-Cerebellar Cortex | 0.162 | 1.404 | 0.41 | 0.007 | 2.754 | 0.945 | 0.139 | 1.489 | 0.532 |
| Right-Cerebellar Cortex | 0.16 | 1.412 | 0.41 | 0.026 | 2.245 | 0.839 | 0.331 | 0.975 | 0.44 |
| Left-Cerebellar White Matter | <0.001 | 3.634 | 0.74 | 0.097 | 1.672 | 0.514 | 0.116 | -1.582 | -0.214 |
| Right-Cerebellar White Matter | <0.001 | 3.819 | 0.735 | 0.268 | 1.113 | 0.398 | 0.023 | -2.305 | -0.359 |
| Left- Cerebral Cortex | 0.001 | 3.377 | 0.738 | 0.036 | 2.122 | 0.946 | 0.367 | -0.904 | 0.051 |
| Right- Cerebral Cortex | 0.001 | 3.305 | 0.718 | 0.019 | 2.372 | 1.019 | 0.555 | -0.591 | 0.126 |
| Left-Cerebral White Matter | <0.001 | 5.837 | 1.09 | 0.096 | 1.675 | 0.846 | 0.001 | -3.548 | -0.434 |
| Right-Cerebral White Matter | <0.001 | 5.554 | 1.021 | 0.074 | 1.802 | 0.924 | 0.002 | -3.169 | -0.336 |
| Brain-Stem | <0.001 | 4.219 | 0.837 | 0.033 | 2.149 | 0.804 | 0.105 | -1.63 | -0.057 |
| Total Cortex | 0.001 | 3.359 | 0.731 | 0.025 | 2.259 | 0.987 | 0.453 | -0.752 | 0.089 |
| Total Cerebral White Matter | <0.001 | 5.721 | 1.059 | 0.083 | 1.746 | 0.889 | 0.001 | -3.373 | -0.386 |
| Total Deep Grey Matter | <0.001 | 6.984 | 1.345 | 0.018 | 2.404 | 1.027 | <0.001 | -3.847 | -0.459 |
| Total Grey Matter | <0.001 | 3.764 | 0.786 | 0.012 | 2.56 | 1.078 | 0.417 | -0.814 | 0.103 |
| Total Intracranial Volume | 0.169 | 1.382 | 0.342 | 0.12 | 1.563 | 0.828 | 0.748 | 0.322 | 0.351 |
| **Deep grey matter** |  |  |  |  |  |  |  |  |  |
| Left-Thalamus | <0.001 | 7.529 | 1.491 | 0.061 | 1.888 | 0.814 | <0.001 | -4.849 | -0.676 |
| Left-Caudate nucleus | <0.001 | 4.957 | 0.988 | 0.056 | 1.924 | 0.761 | 0.013 | -2.514 | -0.333 |
| Left-Putamen | <0.001 | 5.594 | 1.089 | 0.065 | 1.858 | 0.884 | 0.002 | -3.149 | -0.422 |
| Left-Globus pallidum | <0.001 | 5.012 | 0.984 | 0.084 | 1.742 | 0.521 | 0.007 | -2.744 | -0.451 |
| Left-Amygdala | <0.001 | 3.667 | 0.728 | 0.168 | 1.386 | 0.782 | 0.06 | -1.897 | -0.088 |
| Left-Nucleus Accumbens | <0.001 | 4.976 | 1.028 | 0.012 | 2.549 | 0.814 | 0.058 | -1.908 | -0.238 |
| Left-Ventral Diencephalon | <0.001 | 5.496 | 1.109 | 0.02 | 2.348 | 0.956 | 0.011 | -2.574 | -0.218 |
| Left-Hippocampus | 0.002 | 3.192 | 0.665 | 0.175 | 1.364 | 0.64 | 0.137 | -1.494 | -0.122 |
| Right-Thalamus | <0.001 | 8.09 | 1.575 | 0.013 | 2.516 | 0.976 | <0.001 | -4.724 | -0.688 |
| Right- Caudate nucleus | <0.001 | 4.707 | 0.985 | 0.092 | 1.696 | 0.665 | 0.013 | -2.517 | -0.334 |
| Right-Putamen | <0.001 | 5.344 | 1.1 | 0.034 | 2.145 | 0.891 | 0.009 | -2.639 | -0.314 |
| Right-Globus pallidum | <0.001 | 4.353 | 0.872 | 0.208 | 1.265 | 0.487 | 0.009 | -2.631 | -0.404 |
| Right-Amygdala | 0.017 | 2.412 | 0.532 | 0.913 | 0.11 | 0.466 | 0.043 | -2.046 | -0.122 |
| Right-Nucleus Accumbens | <0.001 | 6.147 | 1.379 | 0.016 | 2.435 | 0.722 | 0.003 | -3.068 | -0.508 |
| Right-Ventral Diencephalon | <0.001 | 3.983 | 0.832 | 0.134 | 1.506 | 0.764 | 0.041 | -2.059 | -0.124 |
| Right-Hippocampus | <0.001 | 4.081 | 0.848 | 0.239 | 1.183 | 0.573 | 0.015 | -2.469 | -0.282 |

Note: HC, healthy controls; RRMS, relapse-remitting multiple sclerosis; NMOSD, neuromyelitis optica spectrum disorder.
