# Supplementary figures and images for: The different damage patterns of short-, middle- and long-range connections between patients with relapse-remitting multiple sclerosis and neuromyelitis optica spectrum disorder
Source: Front Immunol. 2022 Dec 2;13:1007335. doi: 10.3389/fimmu.2022.1007335 (PMC9755727; doi:10.3389/fimmu.2022.1007335)

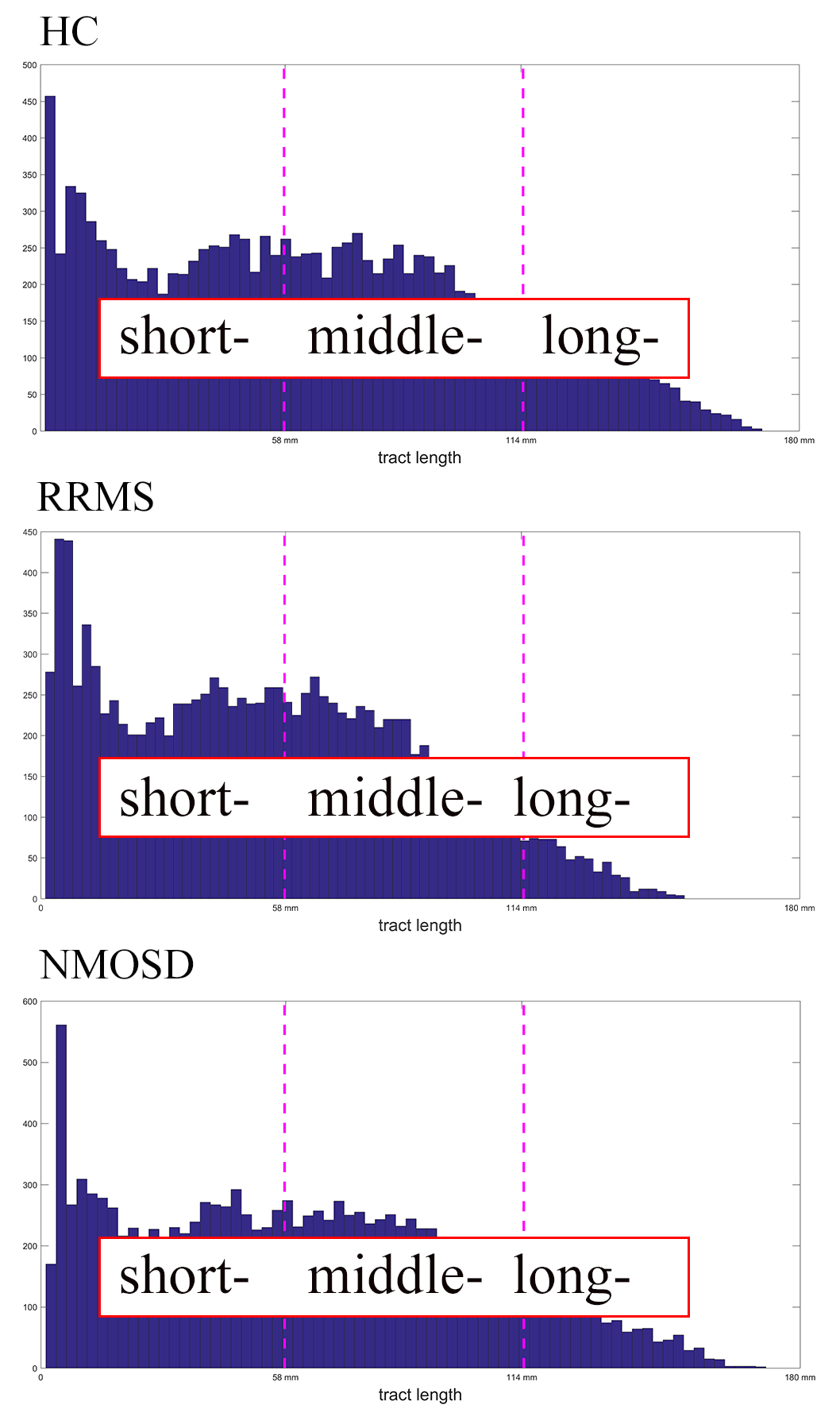

Supplement: Supplementary Figure 1 — The distribution of short-, middle-, and long-range connection in HC, RRMS and NMOSD patients. [file Image_1.png]

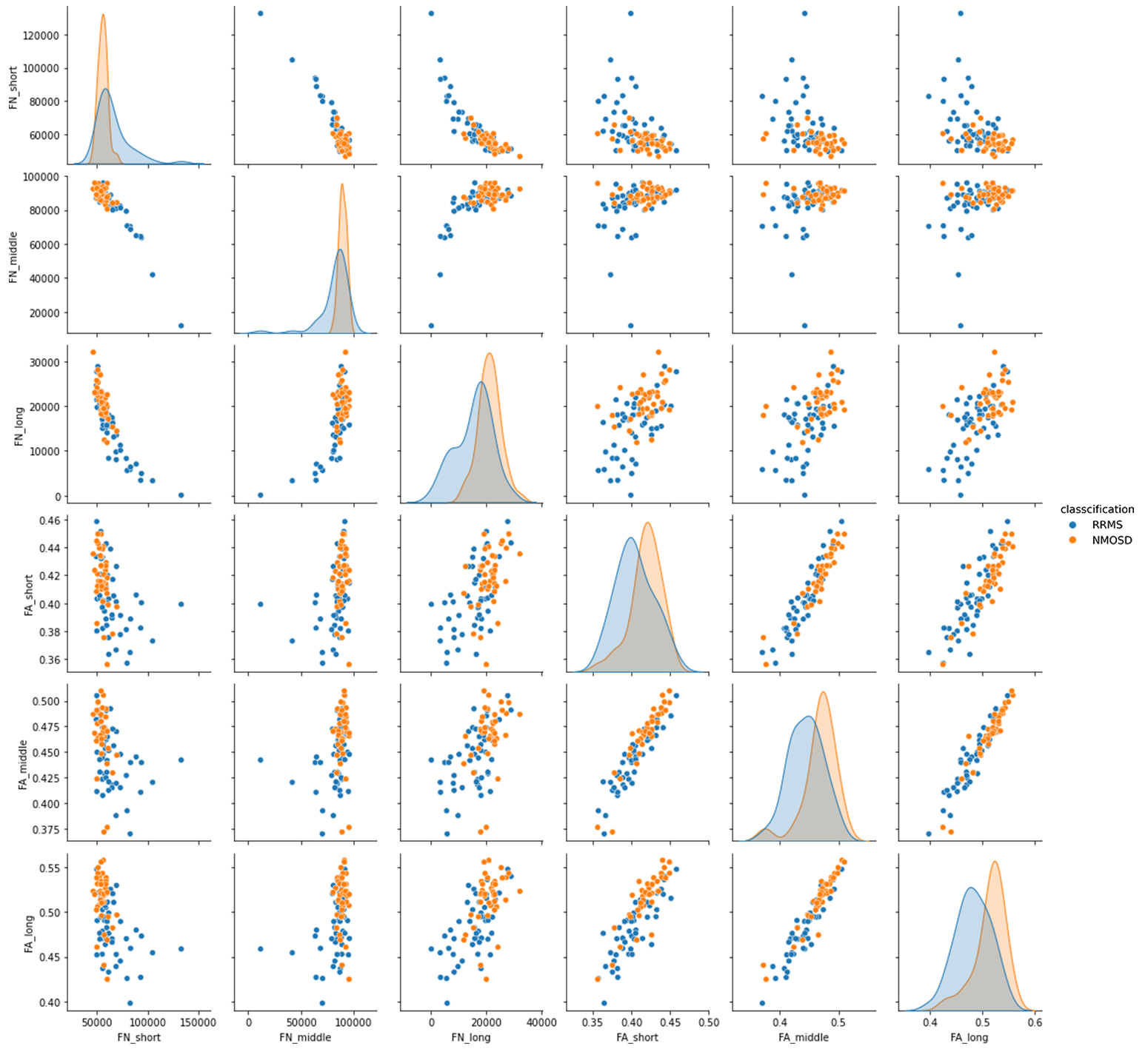

Supplement: Supplementary Figure 2 — The relationships about FN and FA of short-, middle- and long-range connection in patients with RRMS and NMOSD. FN, fiber number; FA, fractional anisotropy. [file Image_2.png]
